# Knowledge gaps on grape sour rot inferred from a systematic literature review

**DOI:** 10.3389/fpls.2024.1415379

**Published:** 2024-07-03

**Authors:** Chiara Brischetto, Vittorio Rossi, Giorgia Fedele

**Affiliations:** Research Center on Plant Health Modelling (PHeM), Department of Sustainable Crop Production (DI.PRO.VE.S.), Università Cattolica del Sacro Cuore, Piacenza, Italy

**Keywords:** *vitis vinifera*, bunch microflora, causal agents, pathogenicity studies, integrated disease control

## Abstract

Sour rot (SR) is one of the major diseases affecting grapevine berries, causing severe yield losses and deterioration of wine quality. SR is caused by an etiologic complex of microorganisms, including yeasts, bacteria, and filamentous fungi. This systematic review focuses on the etiology, epidemiology, and control of SR. A total of 74 papers published between 1986 and 2023 were assessed in this review. Description of disease symptoms was quite consistent across the papers, including oxidation of the grape skin, disaggregation of the internal tissues, and detachment of the rotten berries from the pedicel. The affected bunches are characterized by the smell of acetic acid and ethyl acetate that attracts fruit flies (*Drosophila* spp.). However, several knowledge gaps and/or inconsistencies were identified with respect to SR etiology, epidemiology, and control. Overall, 146 microorganisms were isolated from the affected berries (44.5% yeasts, 34.3% bacteria, and 21.2% filamentous fungi); however, the selected papers could not definitively clarify which species are primarily involved in the etiology of the disease. A general inconsistency was also observed in the methods used to assess the incidence and severity of SR in vineyards, making inter-study comparisons extremely challenging. Inconsistencies were also found in the methods used for pathogenicity assessment in artificial inoculation studies. Furthermore, gaps were detected in terms of SR epidemiology, with a focus on environmental conditions affecting the disease development. The SR management options are limited, and efficacy trials often result in poor, variable, and inconsistent levels of control, which might be attributed to the lack of knowledge on disease epidemiology. These knowledge gaps and inconsistencies were analyzed in this review to inform future research activities.

## 1 Introduction

Sour rot (SR) is one of the late-season, non-Botrytis rots affecting grapevines (Hewitt, 1974; Stapleton and Grant, 1992; Duncan et al., 1995). These rots include Alternaria rot (caused by *Alternaria* spp.), Aspergillus rot (or black sooty mold, caused by black Aspergilla and particularly by *Aspergillus carbonarius*, a toxigenic species producing Ochratoxin A), bitter rot (caused by *Greeneria uvicola*), and others caused by *Penicillium*, *Cladosporium*, and *Rhizopus* spp (Kassemeyer and Berkelmann-Löhnertz, 2009; Loureiro et al., 2012; Steel et al., 2013; Barata, 2011; Hewstone et al., 2007).

Previously, SR was regarded as the final stage of Botrytis bunch rot (BBR) (Bisiach et al., 1986). However, today, it is considered a separate disease (Rooney-Latham et al., 2008; McFadden-Smith et al., 2015; Haviland et al., 2017). Hall et al. (2018a), while investigating the potential competition between the development of BBR and SR on grape clusters, showed that the progression of Botrytis infection and the rate of its colonization halted in the presence of SR.

The etiology and epidemiology of SR are not completely understood (Guerzoni and Marchetti, 1987; Nigro et al., 2006). In the last two decades, however, the disease has gained attention due to its potential to reduce crop yield and wine quality (Nigro et al., 2006; Oriolani et al., 2009; Huber et al., 2011; Barata et al., 2011a, b; Wei et al., 2015). SR is caused by a complex of microorganisms, including yeasts, bacteria, and filamentous fungi (Barata, 2011; Hewstone et al., 2007; Steel et al., 2013). The role of each of these microorganisms in the development of the disease is unclear, making its management challenging and often ineffective (Hall et al., 2018a).

The current systematic review aims at performing a comprehensive analysis of SR, focusing on its etiology, epidemiology, and control. It also intends to identify the knowledge gaps in the research to date to inform future studies.

## 2 Materials and methods

A systematic literature review is a scientific method to identify available literature on a particular topic, which is robust, rigorous, objective, and transparent (Randall and James, 2012). A systematic literature search was undertaken on October 17, 2023, using the following three relevant bibliographic databases: i) Scopus (https://www.scopus.com/); ii) Web of Science Core Collection (http://webofknowledge.com/WOS); and iii) Google Scholar (https://scholar.google.it/).

Searches were conducted in English, and the search terms “sour rot,” “grape,” and “*vitis vinifera*” were combined into search strings using wildcards and connectors. The wildcard (*) enables the search to detect multiple word endings. For example, “grape*” would detect grape, grapes, and grapevine. Search terms were combined using the operators AND (both terms must be present somewhere in the search field) and OR (at least one of the terms must be present in the search field). Specific queries were formulated to search academic papers, reviews, papers in press, conference papers, and Ph.D. dissertations. The search was restricted to titles, abstracts, and keywords in Scopus and Web of Science, and titles in Google Scholar.

The following search strings were used: Scopus: (TITLE-ABS-KEY ((“sour rot”) AND (grape* OR “*vitis vinifera*”))) – 79 hits; Web of Science: (TS= ((“sour rot”) AND (grape* OR “*vitis vinifera*”))) – 121 hits; Google Scholar: (allintitle: “sour rot” grape OR “*vitis vinifera*”) – 32 hits.

**Figure 1:**
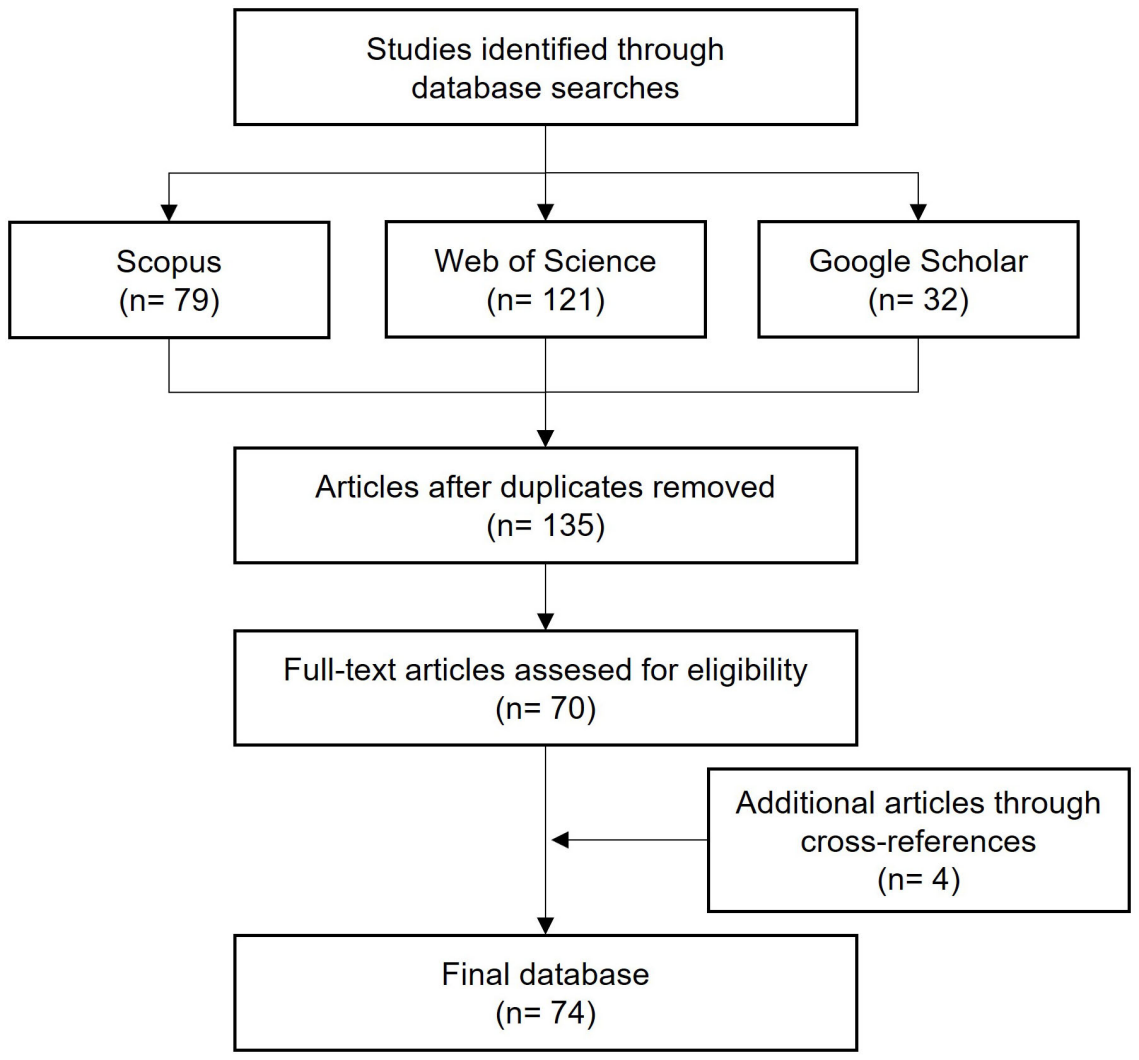
Schematic representation of the systematic review process [based on Biesbroek et al. (2013) and Mckenzie and Joy (2020)].

The papers identified through the search were merged, and duplicates were removed to obtain a refined list comprising 135 papers (**Figure 1**). These papers were then screened based on the following criteria: i) The name of the disease appears in the title, abstract, and keywords; ii) The disease affects *vitis vinifera* ; and iii) The paper concerns the epidemiology, etiology, or control of SR. Abstracts of these papers were read using the RefWorks software (ProQuest, MI, USA), and papers not fulfilling the inclusion criteria were discarded. Through this approach, 70 papers were shortlisted. After examining the bibliography of the shortlisted papers, we identified and included four more papers to the list of shortlisted papers.

Next, we extracted relevant data from these 74 papers based on a structured scheme comprising the following items: (i) Bibliographic information: Author(s), title, and year of publication; (ii) Location of the study (country); (iii) Grapevine varieties included in the study; iv) SR symptoms and methods for disease assessment (e.g., visual observations or chemical analysis); (v) Microorganisms associated with SR in vineyards (i.e., microorganisms isolated from symptomatic berries in field studies); vi) Microorganisms used for artificial inoculation studies; vii) Epidemiological information (e.g., vectors, environmental conditions, etc.); and vi) SR management approaches adopted and their efficacy.

## 3 Results

### 3.1 Bibliographic information

**Figure 2:**
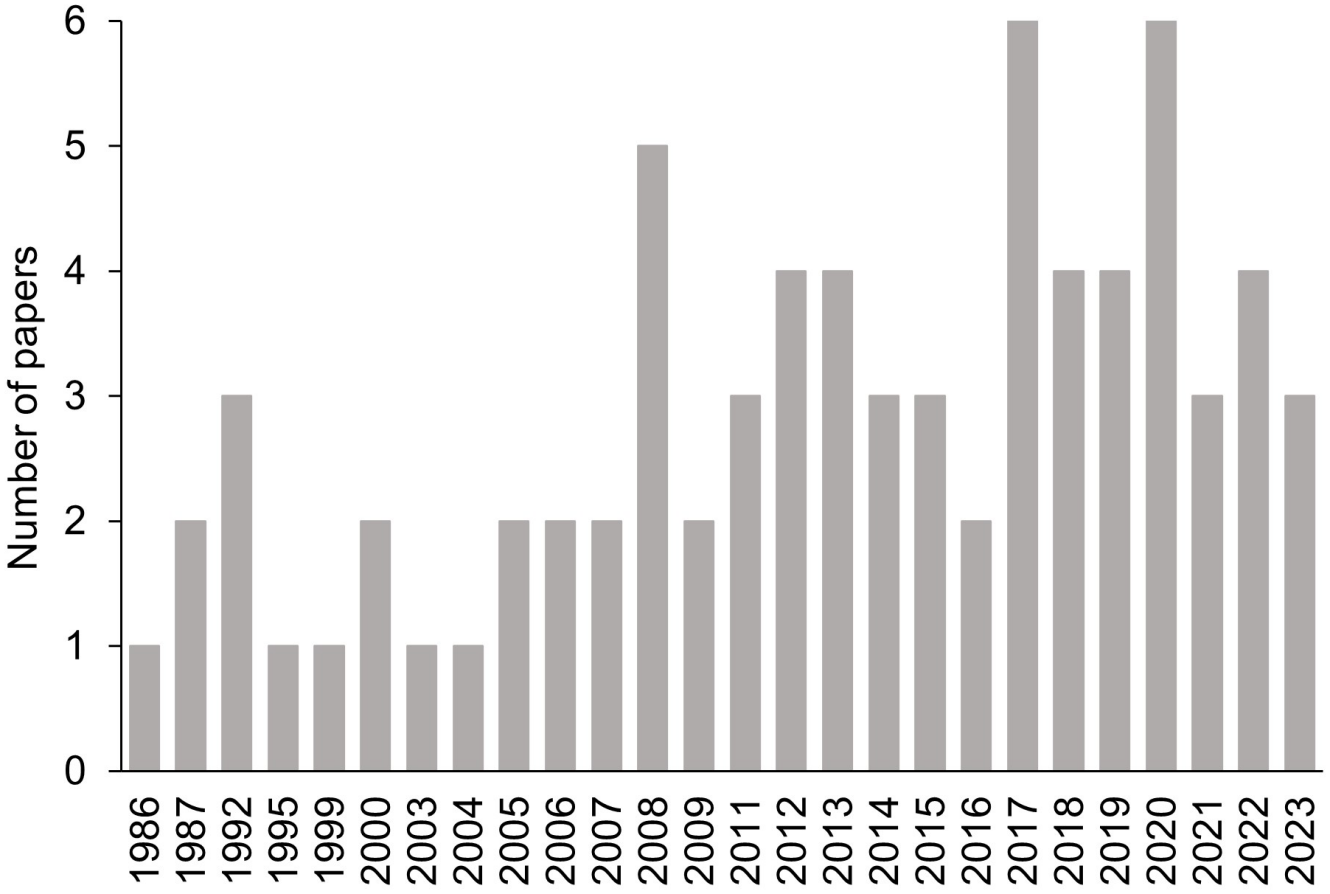
Number of papers published per year.

**Figure 2** shows the number of shortlisted papers published per year. The earliest paper was published in 1986 by Bisiach et al. (1986). From 1995 to 2007, only one or two papers were published each year. Thereafter, the number of publications increased each year. The highest number of publications per year (n = 6) were obtained from 2017 and 2020. The trend shows the increasing interest in SR in the last two decades.

### 3.2 Location of the studies

**Figure 3:**
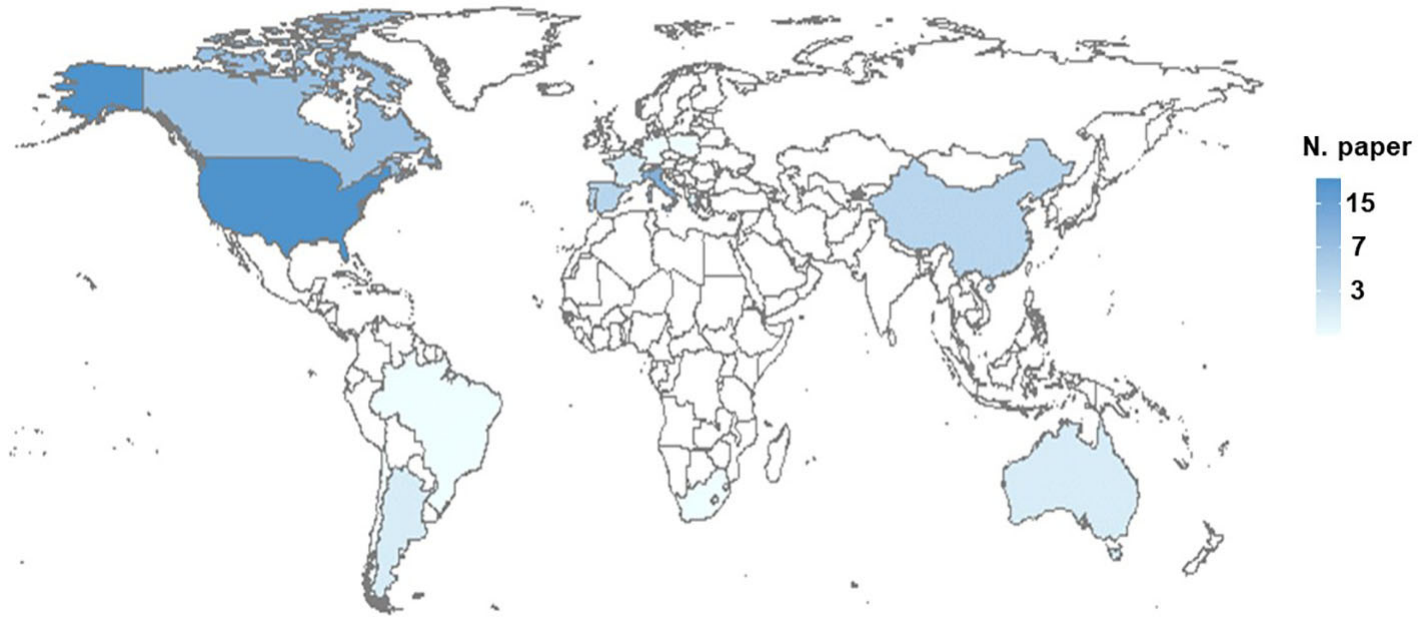
Distribution of sour rot studies worldwide. The total number of papers per country is shown as a color gradient, from white (no papers) to dark blue (high number of papers).

**Figure 3** shows the worldwide distribution of the papers. The papers spread across a total of 16 countries. The highest number of papers were conducted in the US (n = 24), followed by Nord and South Italy (n = 13), Canada (n = 7), China (n = 4), Portugal (n = 4), Spain (n = 4), and South America (n = 3). Eight papers were conducted in France, Greece, Australia, and Israel (each n = 2). Eight papers were conducted in Germany, Poland, and South Africa (each n = 1). Furthermore, more papers were conducted in the northern than in the southern hemisphere (n = 65 vs. 9, respectively).

### 3.3 Grapevine varieties included in the studies

The selected papers mentioned a total of 118 grape varieties, with 59 white (n = 22 table grape; n = 37 wine grape) and 59 red grape varieties (n = 19 table grape; n = 40 wine grape) (**Table 1**). The most frequent cultivars were Sauvignon Blanc and Vignoles (n = 9), followed by Riesling, Pinot noir, Red Globe, Thompson Seedless (n = 7), and Chardonnay (n = 6). It is not clear whether these cultivars were included in the studies due to their relevance in the study area or their level of susceptibility to SR. However, it is commonly accepted that late-ripening cultivars with thin-skinned and tightly packed clusters (e.g., Vignoles, Riesling, Pinot Noir, Pinot Gris, Seyval, etc.) are particularly susceptible to rots close to harvest (Barata et al., 2012a; McFadden-Smith et al., 2015; Bordelon, 2016) because they are prone to berry splitting, and microclimatic conditions within the cluster are more conducive to SR (Steel et al., 2016; Lisek and Lisek, 2021; Cornelissen et al., 2023). Fidelibus et al. (2006, 2007, and 2009) evaluated different varieties of Chardonnay, Merlot, and Barbera and observed that the varieties with larger berries were more susceptible to SR, as large berries often contribute to the development of tight clusters. Lisek and Lisek (2021) assessed the susceptibility to SR of 28 wine and 25 table grape cultivars with diverse geographic and genetic origins. These cultivars were characterized by a significant variation in the density of bunches (loose to very dense), skin thickness (thin to thick), and the time of veraison. They also showed that wine cultivars such as Riesling, Pinot Noir, and Seyval had the highest susceptibility to SR due to their dense clusters and thin skin. Among table grape cultivars, Rusven, Piesnia, Krasotka, Galbena Nou, and Argo, which originated from Russia and Ukraine, exhibited the highest susceptibility to SR (Lisek and Lisek, 2021).

**Table 1:** Grape varieties discussed in the selected papers, with the numbers in parentheses depicting the numbers of cultivars.

**Table** **Grapes**	**White**	Argo, Arkadia, Aron, Bical, Chasselas Dore, Evita, Galbena Nou, Garantos, Krasotka, Palatina, Perlette, Piesnia, Reliance, Rusven, Somerset Sdl, Sophie, Sublima, Suzi, Vostorg (1); Continental Seedless (2); Delight (3); Thompson Seedless (7)
	**Red**	Alden, Antracyt, Beniizu, Festivee, Galanth, Kyoho, Midnight Beauty Muscat Bleu, Muscat Hamburg, Nero, NY Muscat, Price, Rizamat, Scarlotta, Valiant, Yatomi Rosa (1); Crimson Seedless, Fraoula (2); Red Globe (7)
**Wine** **Grapes**	**White**	Brianna, Chasselas, Cococciola, Colombard, Felicia, Frontenac, Hanepoot, Hibernal, Inzolia, Johanniter, La Crosse, Macabeo, Petit Manseng, Phoenix, Roussanne, Sémillon, Seyval, Siegerrebe, Silvaner, Solaris, Souvignier Gris, Tinta Cão, Tocai Friulano, Traminer Rot, Veltliner Frührot, Villaris, Viognier (1); Italia, Muscat, Pinot Blanc (2); Chenin Blanc, Pinot Grigio (3); Chardonnay (6); Riesling (7); Sauvignon Blanc, Vignoles (8)
	**Red**	Agiorgitiko, Barbera, Cabernet Cantor, Cabernet Cortis, Cabernet Franc, Calandro, Canonazo, Domina, Fredonia, Gamay, Marechal Foch, Marquis, Monastrell, Montepulciano, Muscat Hamburg, Nero d’Avola, Periquita, Petit Verdot, Pinot Meunier, Primitivo, Prior, Raboso, Reberger, Regent, Rondo, Rossiola, Schiava, Shiraz, Tauberschwarz (1); Cabernet Sauvignon, Carignane, Trincadeira Preta, Zinfandel (2); Dornfelder, Red Muscat, Sangiovese, Uva d’oro (3); Grenache Rouge (4); Merlot (5); Pinot noir (7)

### 3.4 SR symptoms and assessment methods

The description of SR symptoms was consistent across all the selected papers. The symptoms usually start from an injury site or where the berry attaches to the pedicel. In both red and white grape varieties, the skin of the affected berries exhibits oxidation, turning brown (Gravot et al., 2001; Hall et al., 2018b), cracking and becoming extremely fragile. Then, the internal berry tissue disaggregates, softens, and exudes from the skin cracks. In addition, rotten berries might detach from the pedicel (Bisiach et al., 1986; Guerzoni and Marchetti, 1987). The name “sour rot” originates from the strong and pungent smell emanating from the rotten berries as a result of the production of several chemical compounds, such as acetic acid, glycerol, ethyl acetate, ethanol, galacturonic acid, acetaldehyde, and gluconic acid. These compounds form during the fermentation of the berry pulp sugars, resulting in the production of ethanol (mainly by yeasts) that is oxidized into acetic acid (mainly by bacteria) (Pinto et al., 2019), or can be directly produced by SR-related microbes (Marchetti et al., 1984; Zoecklein et al., 2000). Yeasts producing ethanol did not seem to induce SR alone and required the activity of bacteria that produced high levels of acetic and gluconic acid and initiated berry fermentation (Barata et al., 2012b).

Among the selected papers, 28 papers discussed an assessment of the SR symptoms. In the majority of these papers (n = 22), the disease was assessed by visual observations in terms of disease incidence or severity. Disease incidence was expressed as the percentage of clusters showing SR symptoms (Duncan et al., 1995; Tjamos et al., 2004; Dimakopoulou et al., 2008; Calvo-Garrido et al., 2013; Calzarano et al., 2019, 2020; Carbó et al., 2019; Vogel et al., 2020; Kenney and Hall, 2021; Owoyemi et al., 2022), as the proportion of clusters having four or more adjoining berries with SR symptoms (Fidelibus et al., 2005, 2006, 2007, 2009), or as the percentage of affected berries over the total number of detached berries (Gao et al., 2020). Disease severity was expressed as the percentage of affected berries (i.e., berries with SR symptoms) over the total berries in a cluster (Tjamos et al., 2004; Calvo-Garrido et al., 2013; Calzarano et al., 2019, 2020; VanderWeide et al., 2020; Vogel et al., 2020; Kenney and Hall, 2021; Carbó et al., 2019), as the percentage of the cluster surface with SR symptoms (Tjamos et al., 2004; Hall et al., 2018b; Vogel et al., 2020; Lisek and Lisek, 2021; Cornelissen et al., 2022), or as an empirical rating (Schena et al., 2005; Nigro et al., 2006; Huber, 2016; Madden et al., 2017; Pinto et al., 2017; Hall et al., 2018b; Calderone et al., 2022).

However, the scales for rating disease severity were not uniform. Schena et al. (2005) used the following scale: 0 = bunch without rots and 1 = 1–5%, 2 = 6–10%, 3 = 11–25%, and 4 = >25% of rotted berries in a cluster. Huber (2016) used the following scale: 0 = 0%, 1 = <10%, 2 = 10–25%, 3 = 25–75%, and 4 = >75% infected tissue. Calderone et al. (2022) used the following scale: 0 = 0%, 1 = 1–25%, 2 = 25.1–50%, 3 = 50.1–75%, and 4 = 75.1–100% of affected berries in a cluster. Nigro et al. (2006) used an empirical scale as follows: 0 = bunch without rots and 1 = 1–5%, 2 = 6–10%, 3 = 11–25%, 4 = 26–50%, 5 = 51–75%, 6 = >76% of rotted berries. Different from other studies, Hall et al. (2018a) rated disease severity through the following scale: 0 = berry is symptomless and completely intact; 1 = berry is completely intact, with some discoloration of the skin only around the wound site; 2 = berry is entirely intact but with obvious discoloration of the skin extending beyond the wound site; 3 = majority of the berry skin is discolored but berry is still intact; and 4 = berry is no longer intact, the inner pulp is liquefied and leaking, and the skin is completely discolored (characteristic SR symptoms). Pinto et al. (2017) evaluated disease severity using an empirical scale with five degrees: 0 = sound berries and 1 = 1–10%, 2 = 11–20%, 3 = 21–50%, and 3 = >50% spoilage of berry surface. Madden et al. (2017) used a qualitative scale based on the images of the affected berries to assess the severity of berry decomposition. The lack of uniformity in the methods for disease severity assessment makes the inter-study comparison difficult, warranting a need for a common standard scale.

In nine papers, the disease was assessed indirectly, based on the changes in the chemical composition and properties of the juice of affected berries in comparison to the juice of healthy ones, including volatile acidity, pH, and levels of acetic acid, ethanol, glycerol, and gluconic acid (Zoecklein et al., 2000; Barata et al., 2012b; Mateo et al., 2014; Hall et al., 2015; Madden et al., 2017; Pinto et al., 2017; Hall et al., 2018a; Pinto et al., 2019; Cornelissen et al., 2022). Glycerol, acetic acid, and gluconic acid levels are considered SR markers (Barata et al., 2012b; Cornelissen et al., 2022). The most commonly used technique for chemical analysis was high-performance liquid chromatography (HPLC) (Zoecklein et al., 2000; Hall et al., 2015; Hall et al., 2018a; Mateo et al., 2014; Pinto et al., 2017, 2019) using a column packed with hydrogen sulfonated divinyl benzene-styrene (Castellari et al., 2000). The advantages of this method include speed, simplicity, accuracy, precision, and sensitivity (Kupina, 1984). Some authors also used respective commercial assay kits (Barata et al., 2012b; Madden et al., 2017; Cornelissen et al., 2022). Notably, the results of visual rating were not consistent with those of chemical analysis (Madden et al., 2017; Hall et al., 2018b). According to Madden et al. (2017), this discrepancy might be attributed to SR-associated filamentous fungi that metabolize organic acids in affected berries. Hall et al. (2018b) observed that the relationship between SR symptoms and acetic acid levels in berries depends on the combination of yeasts and acetic acid bacteria (AAB) they harbor. Hence, it is difficult to compare the results of studies that assessed disease severity solely on the basis of symptoms or the chemical composition of berries.

### 3.5 SR-associated microbes in vineyards

The surface of grape berries harbors a complex microbial community comprising filamentous fungi, yeasts, and bacteria (Barata et al., 2012c; Rousseaux et al., 2014). Among the selected papers, 22 papers focused on isolating and identifying the microbes present on the berry surface and 10 of these papers compared the surface microbial diversity between SR-affected and -unaffected berries.

In these studies, the microorganisms were isolated by plating a suspension obtained by washing the berry surface or berry juice collected in the vineyards onto the following artificial media: i) General yeast peptone (GYP) agar (Barata et al., 2011b) for yeasts; ii) selective/differential media for specific microorganisms (Schuller et al., 2000); iii) glucose yeast extract (GY) and De Man, Rogosa, and Sharpe (MRS) agar (De Man et al., 1960) for enumeration of AAB and lactic acid bacteria (LAB), respectively; and iv) Czapeck-Agar medium for fungi. After isolation, the microorganisms were identified based on the morphology of colonies and/or the sequencing of standard rDNA regions, i.e., the 5.8S-internal transcribed spacer (ITS) for yeast and fungi, and the 16s ribosomal RNA (rRNA) for bacteria.

**Figure 4:**
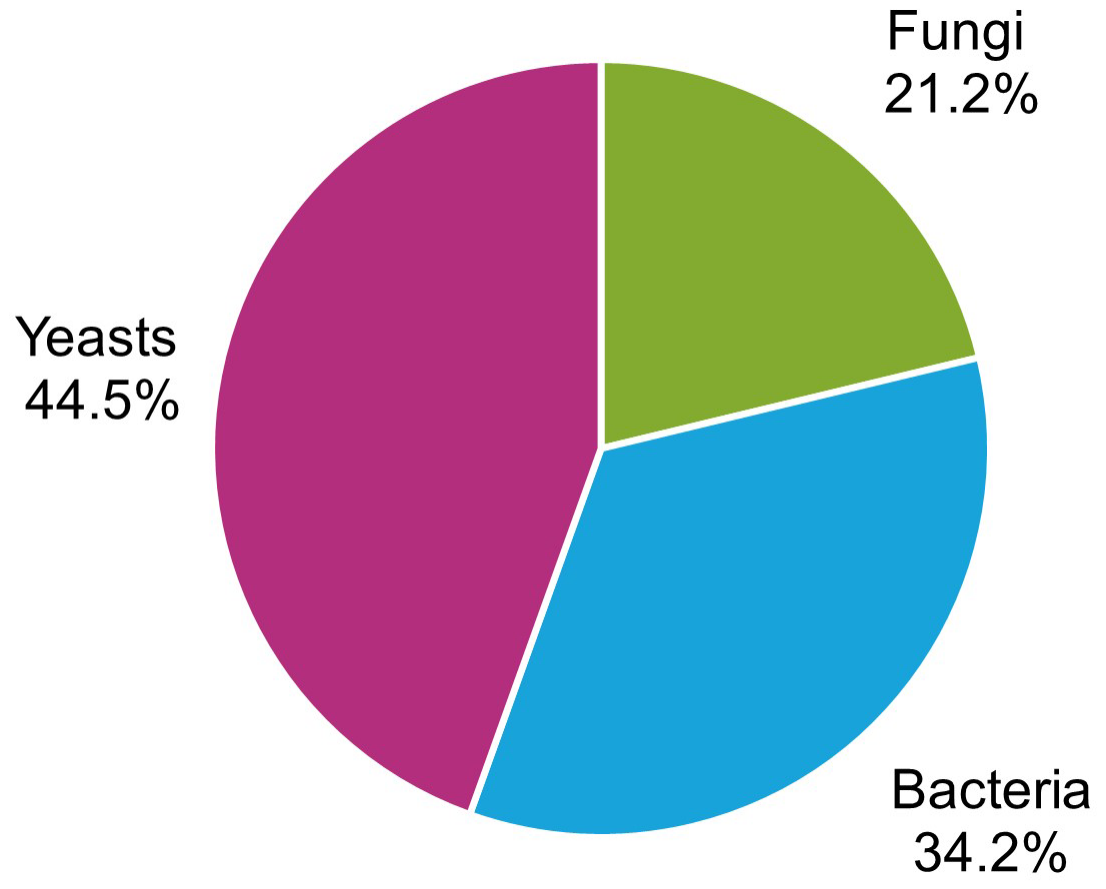
Frequency of the microorganisms isolated from grape bunches affected by sour rot, grouped as yeasts, bacteria, and filamentous fungi.

**Figure 5:**
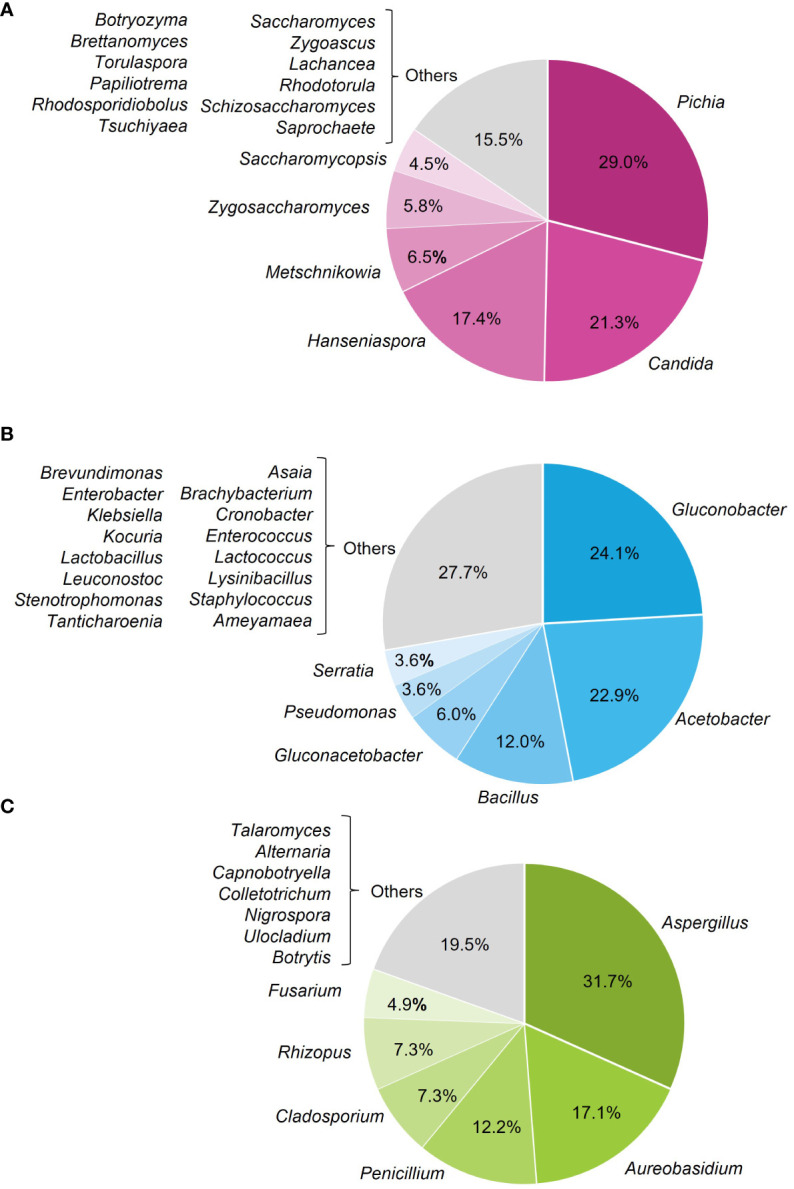
Frequency of the microorganisms isolated from grape bunches affected by sour rot, based on genera of yeasts **(A)**, bacteria **(B)**, and filamentous fungi **(C)**.

Overall, 146 microorganisms were isolated from the affected berries, with 44.5% yeasts, 34.3% bacteria, and 21.2% filamentous fungi (**Figure 4**). The most frequently isolated genera were *Pichia* (29.0%), *Candida* (21.3%), and *Hanseniaspora* (17.4%) among yeasts (**Figure 5A**); *Gluconobacter* (24.1%), *Acetobacter* (22.9%), and *Bacillus* (12.0%) (**Figure 5B**) among bacteria; and *Aspergillus* (31.7%) and *Aureobasidium* (17.1%) among filamentous fungi (**Figure 5C**).

While AAB were commonly associated with SR (Barata et al., 2012a, b), there is some debate about some yeast species. For example, Barata et al. (2012b) showed that some yeast species (i.e., *Pichia terricola*, *Hanseniaspora uvarum*, and *Candida zemplinina*) were not able to produce the metabolites characteristic of SR (gluconic and acetic acids) in inoculated berries. In contrast, Huber (2016) showed that *H. uvarum* and *C. zemplinina* contributed to the development of SR symptoms, in accordance with other literature (Gravot et al., 2001).

Some authors reported the presence of *Penicillium*, *Cladosporium*, *Alternaria*, and *Rhizopus* members in SR-affected berries (Bisiach et al., 1986; Hewstone et al., 2007). However, Bisiach et al. (1986) and Oriolani et al. (2007) stated that these fungi were not capable of initiating an SR infection and considered these fungi as endophytes, secondary invaders, or producing quiescent infection in berries (Duncan et al., 1995; Mostert et al., 2000; Dugan et al., 2002; Barata et al., 2008; González and Tello, 2011; Steel et al., 2013). In contrast, Latorre et al. (2002) reported that *Aspergillus* spp., *Penicillium* spp., *Rhizopus* spp., and *Cladosporium* spp. were primarily responsible for SR in Chile, with yeasts and bacteria being secondary invaders. In addition, Rooney-Latham et al. (2008) found that *A. niger* and *A. carbonarius* were the first microbial species to colonize the wounded berries and initiate their rot. Following Wilcox et al. (2015), these fungi and namely Aspergilla, do not cause true SR even though AAB can produce the vinegar smell in rotten berries; it would be desirable to have a common disease definition of the (true) SR, as a disease characterized by the rapid decomposition of ripening grape berries which exude the vinegarlike odor of acetic acid and ethyl acetate, which is caused by yeasts and AAB.

**Figure 6:**
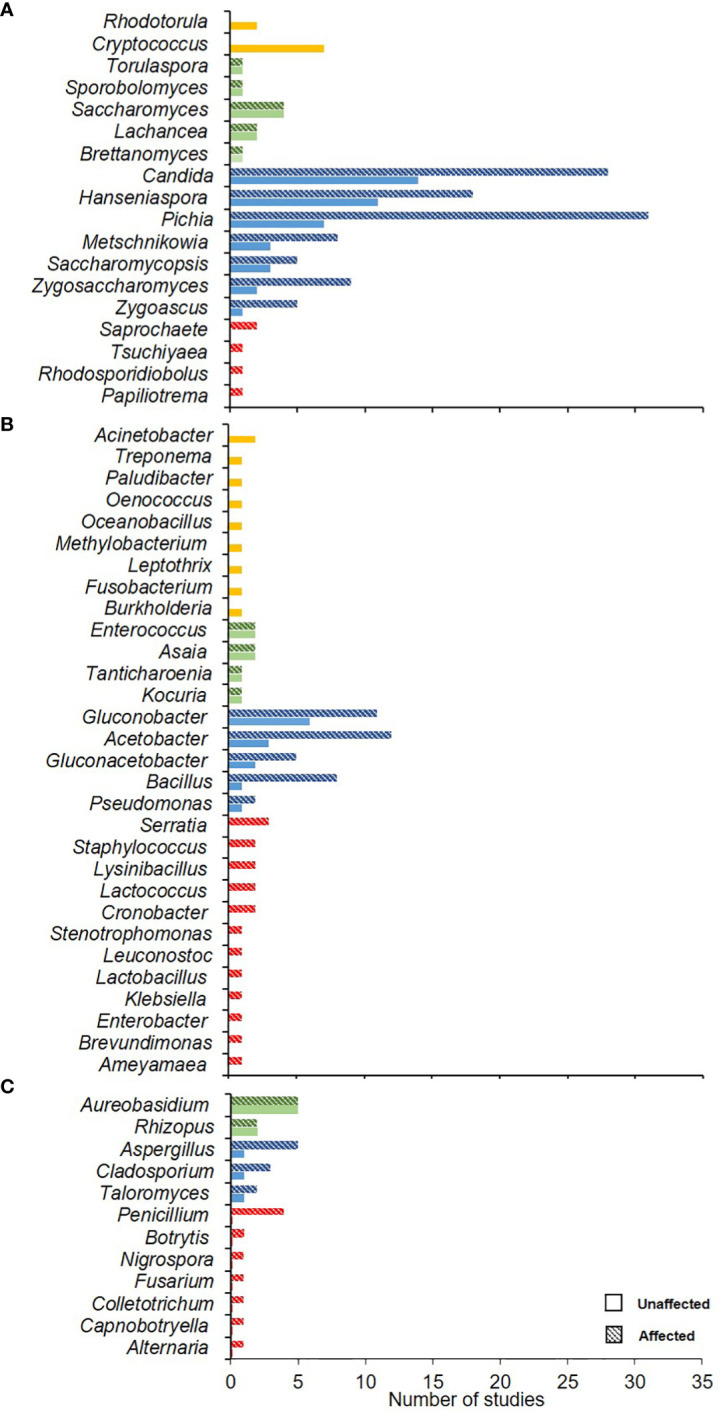
Number of studies wherein each microorganism (yeasts, **A**; bacteria, **B**; and filamentous fungi, **C**) was isolated from grape bunches unaffected and affected by sour rot. Yellow and red colors represent the microorganisms found only on unaffected and affected bunches, respectively. Green and blue colors represent the genera found in unaffected (full bars) and affected (dotted bars) bunches, in equal or different numbers, respectively.

When the microorganisms from SR affected and un-affected berries were compared in the same vineyard, there were 69 microorganisms isolated from un-affected berries and 128 from affected ones. Among the filamentous fungi, no significant difference was observed in the frequencies of *Aureobasidium* and *Rhizopus*, while *Aspergillus*, *Cladosporium*, and *Taloromyces* were more frequently isolated from the affected berries (**Figure 6A**). Among the bacteria, *Gluconobacter*, *Acetobacter*, *Gluconoacetobacter*, *Bacillus*, and *Pseudomonas* were more prevalent in the affected berries (**Figure 6B**). Among the yeasts, *Saccharomyces* was frequently isolated from both unaffected and affected berries, while *Pichia* and *Candida* were more prevalent in the affected berries (**Figure 6C**). These comparisons revealed that some microbes were more prevalent in the affected berries. However, the metadata from these studies did not allow a statistical comparison between the affected and unaffected berries. Thus, these studies could not determine which species are primarily involved in SR etiology.

The abundance and diversity of microbial species found in the different studies vary based on grape varieties, grape ripening stages, environmental conditions, soil types, cultivation practices, and the general grape health status (Barnett et al., 1972; Davenport, 1974; Rosini et al., 1982; Longo et al., 1991; Yanagida et al., 1992; Schütz and Gafner, 1994; Martini et al., 1996; De La Torre et al., 1999; Guerra et al., 1999; Mortimer and Polsinelli, 1999; Pretorius et al., 1999; Fleet et al., 2002; Combina et al., 2005; Valero et al., 2005; Raspor et al., 2006; Nisiotou and Nychas, 2007; Valero et al., 2007; Chavan et al., 2009; Li et al., 2010; Rousseaux et al., 2014). These factors significantly influence the composition of the grape microbiome (Raspor et al., 2006) and, therefore, the prevalence of some species over others. For example, *Aureobasidium pullulans* was isolated more frequently from red than white grapevine cultivars (Raspor et al., 2006). Moreover, a greater yeast population was found in Merlot than in Cabernet Sauvignon berries (Renouf et al., 2005). In China, the yeast population density and diversity of three grape varieties varied across different vine-growing regions (Li et al., 2010), with *H. uvarum* being rarely found in regions with cool and dry climates.

The availability of nutrients in grape berries may also influence the composition and size of epiphytic microbial populations (Shiraishi et al., 2010). The presence of (micro)cracks or wounds on the berry skin increases by about 100 times the content of sugars, organic acids, and amino acids on the surface of berries in comparison to intact berries, enabling a massive growth of opportunistic microorganisms (Fonseca and Inácio, 2006; Loureiro et al., 2012). In addition, the interactions between the microorganisms present on the surface of affected berries can influence the composition of the microflora. For instance, the presence of acetic acid due to AAB can inhibit the growth of the yeast population and slow down the accumulation of ethanol deriving from soluble sugar metabolism (Pinto et al., 2019).

### 3.6 Artificial inoculation studies

In eight of the selected papers, unaffected and intact berries from the vineyard were artificially inoculated with single or multiple microorganisms. The berries were first rinsed in water, then surface disinfected, washed again to remove disinfectants, and finally wounded with a sterile needle. The inoculation methods varied across the studies, including immersion of wounded berries in an inoculum suspension (containing 10^6^–10^7^ cells/mL), spraying of the inoculum suspensions (containing 10^5^–10^7^ cells/mL) on the surface of wounded berries, or pipetting a microbial suspension (containing 10^4^–10^8^ cells/mL) into the wound. Following inoculation, berries were kept at temperatures ranging between 22 and 27°C, in the dark or with a 12-h photoperiod, for 5–14 days, depending on the study.

Furthermore, variability in the methods used for artificial inoculation might have influenced the results of different studies, making them difficult to compare. For instance, Barata et al. (2012b) did not obtain any symptoms of SR even after 12 days of incubation post-yeast inoculation. However, Huber (2016) observed a mean SR incidence of 60% after 14 days of incubation. The two studies used similar concentrations of inoculum (10^6^ and 10^7^ cells/mL) and incubation temperatures (22 and 25°C), but the former study inoculated wounded berries through immersion in the inoculum suspension, while the latter inoculated through pipetting the microbial suspension into the wound.

**Figure 7:**
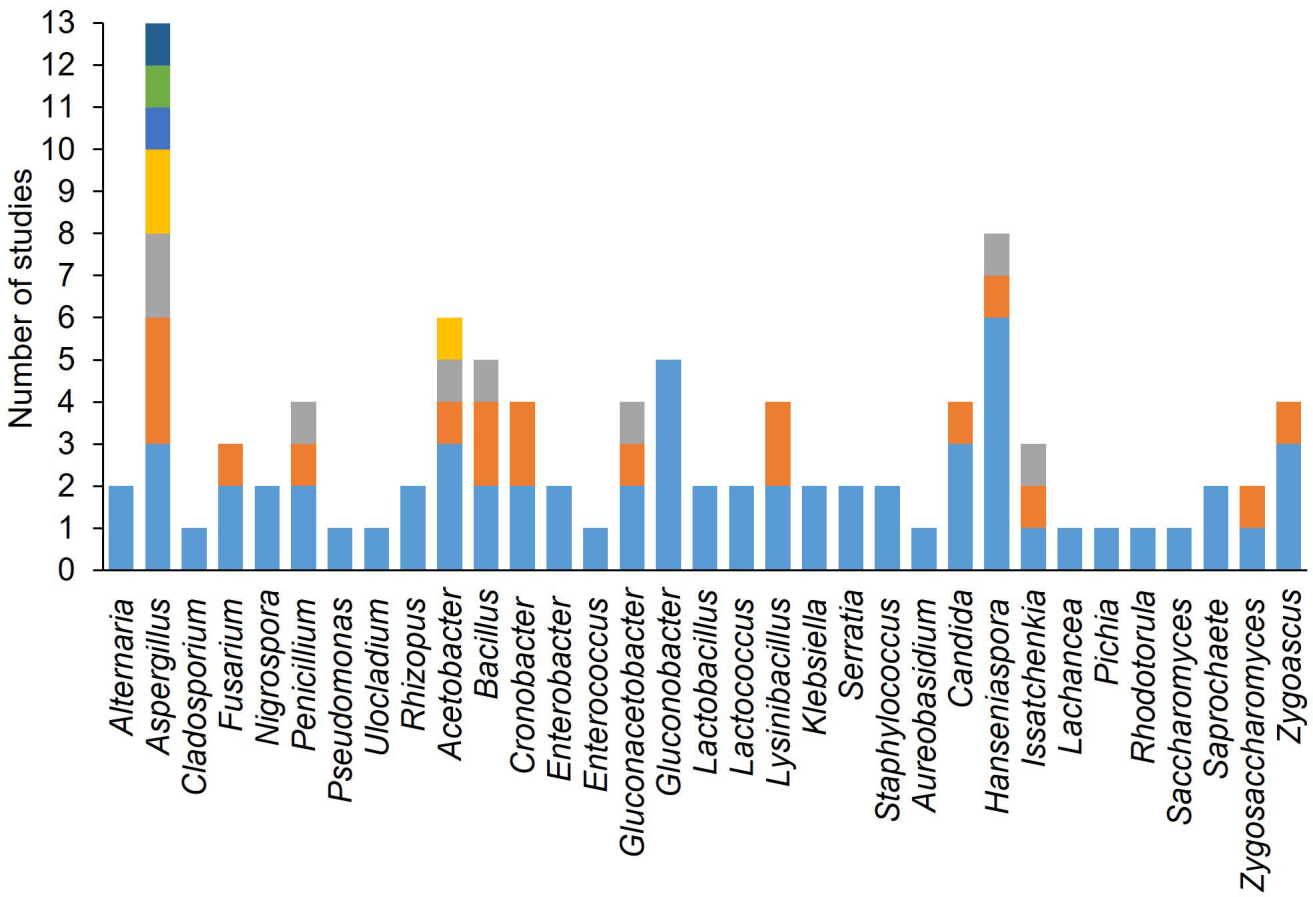
Number of studies wherein each microorganism was used in artificial inoculation studies on berries. The colors of the bars indicate different species within a genus.

**Figure 8:**
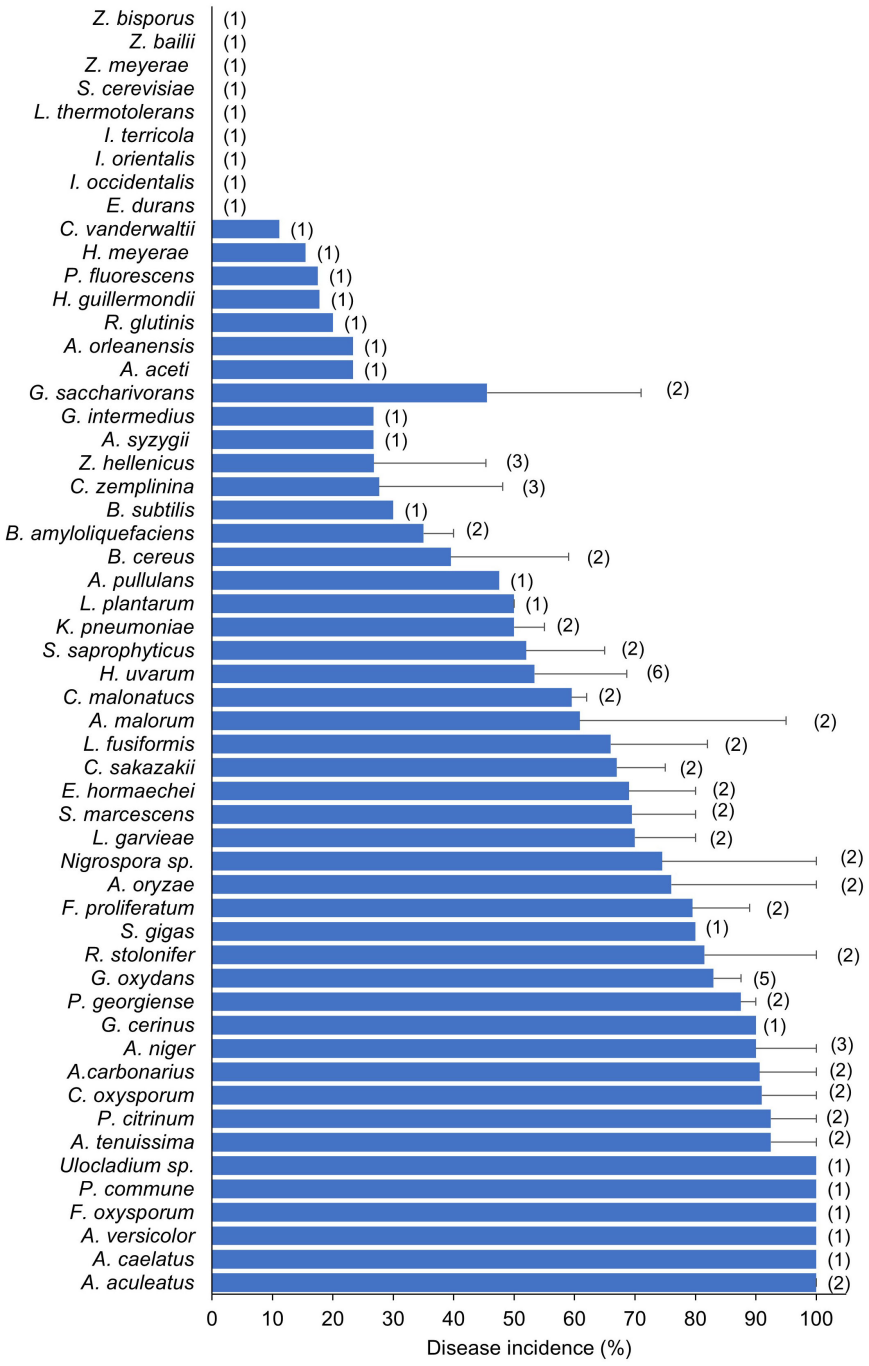
Sour rot incidence in artificial inoculation studies on berries. Bars represent average disease incidence with standard errors; numbers in parentheses indicate the number of studies (that corresponds to the number of entries as disease incidence values used to calculate averages and standard errors).

**Figure 7** shows the frequency with which different yeast, fungal, and bacterial genera were used to inoculate wounded berries. The most frequently used genera were *Aspergillus* (n = 13), followed by *Hanseniaspora* (n = 8), *Acetobacter* (n = 6), *Gluconobacter* (n = 5). *Candida* (n = 4), and *Zygoascus* (n = 4). The most commonly used species were *H. uvarum* (n = 6), followed by *C. zemplinina* (n = 3), *Zygoascus hellenicus* (n = 3), *A. carbonarius* (n = 3), *Acetobacter aceti* (n = 3), and *Gluconobacter oxydans* (n = 5). **Figure 8** shows the mean SR incidence after inoculation of wounded berries with a single species. Moreover, the berries inoculated with *A. aculeatus*, *A. carbonarius*, and *A. niger* showed an SR incidence of 90%. Inoculation with *A. malorum* and *H. uvarum* resulted in 50–60% disease incidence, while inoculation with *C. zemplinina*, *Z. hellenicus*, and *G. saccharivorans* resulted in a lower SR incidence (24 to 28%).

**Figure 9:**
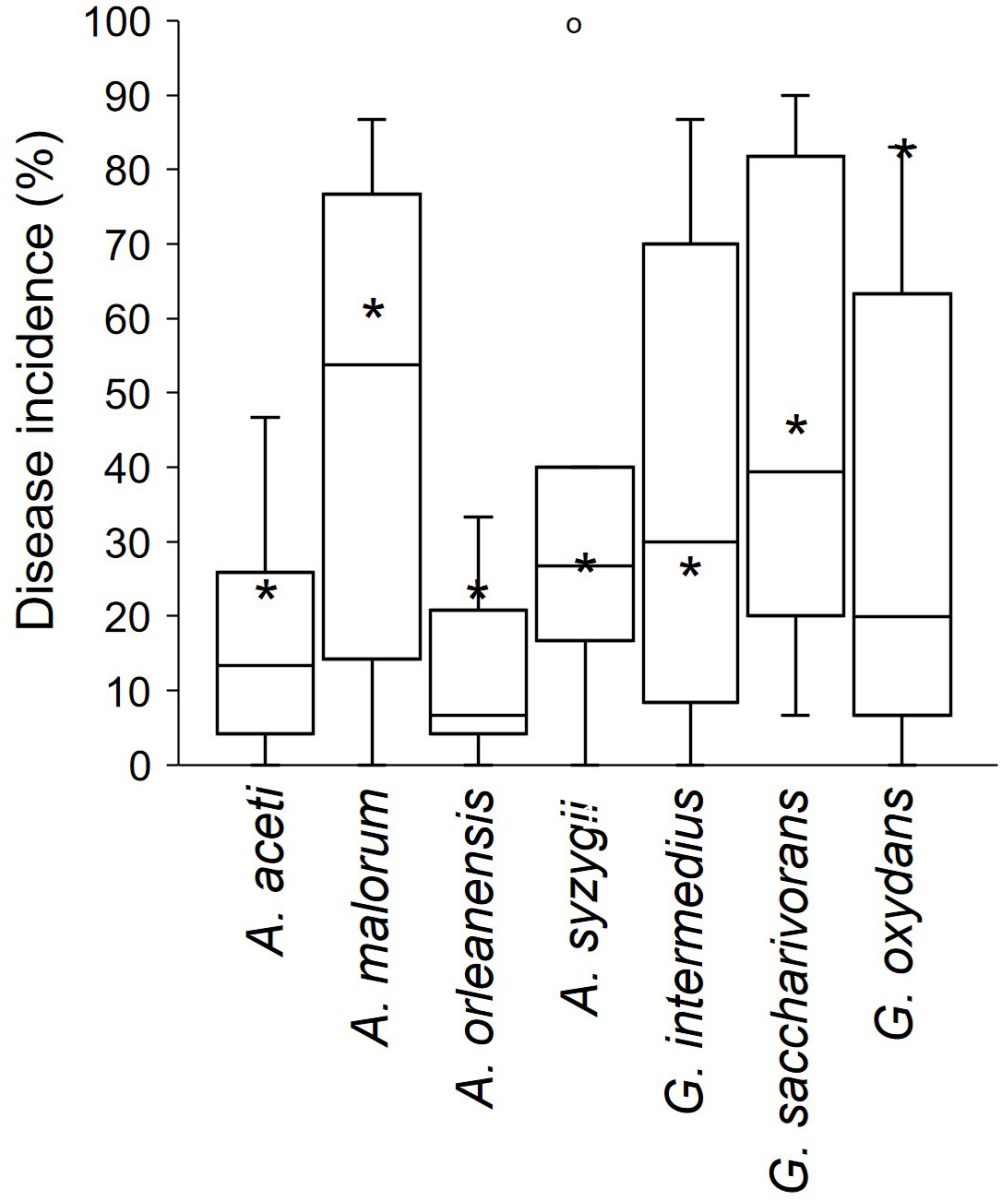
Incidence (%) of sour rot in grapevine berries artificially inoculated with single bacterial species or with consortia of yeasts. (*) indicates the average values of disease incidence in berries artificially inoculated with single bacterial species (data extracted from the whole literature); boxplots represent the artificial inoculation of each single bacterial species with the consortia composed by the following species: Candida vanderwalti, C. zemplinina, Hanseniaspora guilliermondii, H. meyerae, H. uvarum, Zygoascus hellenicus, and Z. meyerae [data provided by Pinto et al. (2017)]. The boxes extend from the 25^th^ to the 75^th^ quartile of the data distribution, the lines crossing the boxes represent the average values, and the whiskers extend to the maximum and minimum values. The dots represent outliers.

The above mentioned studies involving artificial inoculation were mainly conducted using single microbial species. Pinto et al. (2017) evaluated the development of SR in wounded berries inoculated with different yeasts, bacteria, and their combinations. **Figure 9** shows the difference between disease incidences following inoculation with single bacterial species and with combined inoculation of bacteria and yeasts. The results showed that the SR incidence obtained after the inoculation of multiple species was different from that obtained after the inoculation of a single species. In some cases, the inoculation of single species, including *G. oxidans*, *A. aceti*, *A. malorum*, and *A. orleanensis*, resulted in a higher disease incidence than after the inoculation of the consortia of species.

The microbes used in artificial inoculation studies have been commonly associated with SR in vineyards (**Figures 6A–C**) and were isolated more frequently from the affected than the unaffected berries (**Figure 7**). Notably, some relevant microbes, more frequently isolated from the affected than the unaffected berries, have been poorly considered for artificial inoculation studies, such as *Bacillus* and *Pichia*, or never considered, such as *Metschnikowia*. Future studies should consider involving these microorganisms in artificial inoculation.

### 3.7 SR epidemiology

The studies suggest that SR-associated microorganisms penetrate the berries through any kind of wound, whether caused by abiotic (e.g., rain, hail, berry abrasion, etc.) or biotic factors (e.g., insects, birds, etc.), including fungal pathogens such as *B. cinerea* (Marchetti et al., 1984) and *Erisiphe necator* (Gadoury et al., 2007). Physiological microcracks of the berry skin, especially those close to the pedicel favor pathogen penetration (Considine, 1982; Gravot et al., 2001). Microcracks occur naturally on ripening berries due to different causes that are not completely understood (Becker and Knoche, 2012; Ramteke et al., 2017). The number of microcracks also depend on the orientation of the berry in the cluster and berry region (stylar scar, cheek, or pedicel end) (Becker and Knoche, 2012).

It is also commonly accepted that the insects belonging to the genus *Drosophila* (Diptera: Tephritidae) are the main vectors of SR-inducing microbes. Among the 74 selected papers in this review, 14.9% of the papers focused on SR vectors. Of these, 72.7% of papers focused on *Drosophila* spp. (both the fruit fly *D. melanogaster* and the spotted wing *D. suzukii*) while 27.3% of studies investigated other possible insect vectors, namely *Lobesia botrana* (Lepidoptera: Torticiae), *Anastrepha fraterculus* (Diptera: Tephritidae), and *Polistes dominulus* (Vespidae).

Both *D. melanogaster* and *D. suzukii* (Entling and Hoffmann, 2020) are attracted by damaged and rotting fruit (Bisiach et al., 1986; Fermaud et al., 2002; Barata et al., 2012b; Huber, 2016; Hall et al., 2018a). They collect and transport yeasts and AAB on their body and deposit them on the berries by direct contact while feeding (Ioriatti et al., 2018). *D. melanogaster* does not have a sharp ovipositor to cause lesions on the grape epidermis, and thus, they can lay eggs only in wounded or rotten grapes (Atallah et al., 2014; Zhu et al., 2023). In contrast, *D. suzukii* infests on healthy berries through their zig-zag ovipositor, creating wounds on fruits and creating oviposition sites for both species. Once eggs are laid, the larvae develop and trigger the decomposition of berries, thereby inducing SR development (Rombaut et al., 2017; Hall et al., 2018a; Entling and Hoffmann, 2020; Zhu et al., 2023).

Barata et al. (2012b) demonstrated the crucial role of *Drosophila* in SR development, inoculating both intact and wounded berries with different microorganisms, including *C. zemplinina*, *H. uvarum*, *Issatchenkia occidentalis* (current name *Pichia occidentalis*), *I. orientalis* (current name *Pichia kudriavzevii*), *I. terricola* (current name *P. terricola*), *Lachancea thermotolerans*, *Saccharomyces cerevisiae*, *Zygoascus hellenicus*, *Zygosaccharomyces bailii*, *Z. bisporus*, *G. saccharivorans*, and *Enterococcus durans*. The study showed that in the presence of adult flies, all these microbes induced SR in berries. In contrast, in the absence of a vector, only the wounded berries inoculated with *Gluconobacter* develop SR. Hall et al. (2018a) showed that only the berries inoculated with the combination of *Pichia kluyveri* with *G. oxydans*, *S. cerevisiae* with *A. aceti*, and *S. cerevisiae* with *G. oxydans*, in the presence of *D. melanogaster*, induced SR symptoms, confirming the results of Barata et al. (2012b). Furthermore, the authors postulated that the role of *Drosophila* goes beyond vectoring because artificial inoculation studies with axenic flies (lacking gut or surface microbiota) showed that *Drosophila* spp. also has a non-microbial contribution in SR development, presumably through their role in promoting the loss of berry integrity. This is caused by at least in part, enzymes released by the larval stage in order to facilitate consumption of the pulp (Gregg et al., 1990; Sakaguchi and Suzuki, 2013).

In other studies, however, artificial inoculation of wounded berries resulted in SR symptoms even in the absence of flies (Pinto et al., 2017).

Machota et al. (2016) showed that South American fruit fly, *A. fraterculus* (Diptera: Tephritidae), might also facilitate the penetration of SR-inducing microorganisms through the wounds caused by their ovipositor or other body parts. Madden et al. (2017) showed that the European paper wasp, *P. dominulus* (Vespidae), harbors the SR-inducing polymicrobial community and is capable of dispersing living microorganisms when foraging, spreading the disease in the absence of other insect vectors. However, Pavan et al. (2014) concluded that the European grapevine moth *L. botrana* (Lepidoptera: Torticiae) does not play a role in the development of SR in vineyards.

Within the affected cluster, SR can also spread via the fermented pulp oozing from the affected berries and dropping onto the underlying berries (Bisiach et al., 1986; Guerzoni and Marchetti, 1987; Barata et al., 2008; Hall et al., 2018a). Splash dispersal or rain falling on the pulp oozing from rotten berries is also a potential dispersal mechanism, even though it has not yet been documented.

The environmental factors influencing SR development have not been fully investigated. Wounded berries inoculated with *H. uvarum* and *G. oxydans* and incubated at different temperatures (5–10, 10–15, 15–20, and 20–25°C) for 7 days showed increasing disease severity at increasing temperatures, with the most severe symptoms at 20–25°C and least severe symptoms at <10°C (Huber, 2016). These results were in agreement with the cardinal temperatures of *H. uvarum* and *G. oxydans* previously reported by Du Toit and Pretorius (2002) and Salvadó et al. (2011). Other studies reported that warm and rainy conditions between veraison and harvest favored SR epidemic under natural conditions (Zoecklein et al., 2000; Calvo-Garrido et al., 2013; Steel et al., 2013; Huber, 2016).

Climate change potentially elevates SR incidence in many of the major grape-growing regions of the world (Steel et al., 2013). In the past few years, overall SR disease severity, especially in Europe, increased with increasing rainfall events during the period between veraison and harvest (Hausinger et al., 2015; VanderWeide et al., 2020; Lisek and Lisek, 2021; Cornelissen et al., 2023). Additionally, climate warming can accelerate SR spread by directly increasing the vector population that transmits the associated pathogens (Canto et al., 2009; Zhu et al., 2023). Notably, the *D. suzukii* population might decrease at temperatures of >25°C, influencing both adult reproduction and larval survival (Zerulla et al., 2017; Krüger et al., 2021; Zhu et al., 2023). However, a temperature range of 25–29°C does not affect the fecundity of adults or viability of larvae in *D. melanogaster* (Schnebel and Grossfield, 1986; Dillon et al., 2007; Min et al., 2021; Zhu et al., 2023), reproducing six times faster at 25°C than at 12°C (Galet, 1999).

Similarly, the development of *B. cinerea* and non-Botrytis rots can be influenced by high temperatures. *B. cinerea* has an optimum temperature range of 20–25°C for growth (Ciliberti et al., 2015) but does not grow and infect grape berries at temperatures over 30°C (Latorre et al., 2002), providing opportunities for the development of SR-inducing yeasts (Calvo-Garrido et al., 2013). For non-Botrytis rots, the optimum temperatures for the growth of the fungi *C. acutatum* and *G. uvicola*, responsible for ripe rot and bitter rot, respectively, are 25 and 35°C, respectively (Steel et al., 2007). Furthermore, the temperature ranges for optimal growth of *P. expansum*, *A. carbonarius*, and *R. stolonifer* are 20–25, 10–40, and 20–30°C, respectively (Esteban et al., 2004; Judet-Correia et al., 2010; Amiri et al., 2011).

### 3.8 SR control

SR management via chemical means is generally considered poorly effective given the low efficacy of pre-harvest fungicide applications (Gravot et al., 2001; Nigro et al., 2006), with a few exceptions. For instance, fludioxinil (but not cyprodinil and carbendazim) application was highly effective when applied to manage *Aspergillus* spp (Tjamos et al., 2004). Similarly, SR was significantly and consistently reduced by approximately 70% after applying combinations of insecticides (such as the spinetoram and zeta-cypermethrin combination to control *Drosophila* spp.) and antimicrobial agents, such as potassium metabisulfite, copper hydroxide, BLAD (banda de Lupinus albus doce), and/or a mixture of hydrogen dioxide and peroxyacetic acid (Hall et al., 2018b).

Because of the poor efficacy of chemicals, agronomical practices are recommended for SR management. These practices aim at creating a less conducive environment by reducing fruit fly infestation, preventing berry damage by biotic or abiotic factors, and managing the canopy to facilitate air movement and reduce the humidity (Bisiach et al., 1986; Stapleton and Grant, 1992; Zoecklein et al., 1992; McFadden-Smith, 2009; Hall et al., 2018b).

Among the papers selected in the current review, 23% of the papers focused on SR control. In these papers, SR incidence in the NT plots ranged from 0.3 to 97.5%, indicating that the control methods had been tested in a wide range of conditions. Of these, 76% of the papers were conducted in the field and 29% in the laboratory. In particular, three papers involved testing for different biological control agents (BCAs). Four papers compared the application of a single BCA with combinations of BCAs with natural substances or chemicals. Three papers employed only agronomical practices, and in only one paper, the agronomical techniques were combined with chemicals. One paper focused on managing SR with only chemicals, comparing the efficacies of fludioxonil, cyprodinil, and carbendazim. Two papers used natural substances, comparing their efficacies with chemicals or with their combinations with the chemicals. The details of the studies are summarized in **Supplementary Material**.

Among the agronomical practices, leaf removal at the fruit zone decreased the incidence and severity of SR through an elevated air movement and penetration of solar radiation through the canopy, reducing the humidity within the fruiting zone. Leaf removal also allows greater penetration of fungicides into the fruit zone (Gubler et al., 1987; Bettiga et al., 1989).

The efficacy of BCAs in managing SR was assessed under laboratory conditions against various species of *Aspergillus* (*A. caelatus*, *A. carbonarius*, *A. niger*, and *A. terreus*). Twenty-one different BCA species primarily belonging to the following genera were used: *Candida*, *Cyberlindnera*, *Debaryomyces*, *Dekkera*, *Issatchenkia*, *Lachancea*, *Kloeckera*, *Kluyveromyces*, *Pichia*, *Saccharomyces*, and *Torulospora*, for a total of 178 cases (that is, one BCA against one pathogen in one experiment). In 91%, 2.8%, and 7.3% of these cases, BCAs showed low, intermediate, and high efficacies (<30%, 30–60%, and >60% SR reduction compared to untreated control), respectively.

Some BCAs, however, showed very high SR-reducing activity. For instance, *I. orientalis* substantially inhibited *A. terreus*, *C. famata*, *D. vanrijiae*, and *K. marxianus*, reducing their growth by 100%, 76%, 77%, and 82%, respectively. Moreover, *S. chevalieri* and *C. rugosa* affected *A. caelatus* growth (both by 65%) (Nally et al., 2013). In some cases, the level of SR control mediated by BCAs was comparable to that mediated by fungicides. For instance, SR control mediated by *A. pullulans* (73%) was similar to that mediated by the fungicide mixture fludioxonil + cyprodinil (69%) (Dimakopoulou et al., 2008).

Previous studies have reported different mechanisms of SR biocontrol, including the (i) inhibition of spore germination, germ tube, and hyphal growth through the production of bioactive metabolites, enzymes (e.g., laminarinases produced by *S. cerevisiae*, *P. membranifaciens*, *C. catenulata*, and others), and volatile organic compounds, and (ii) competition for nutrients (carbon sources and/or iron) (Nally et al., 2013). For instance, *S. cerevisiae*, *T. delbrueckii*, *C. sake*, *D. vanrijiae*, *C. catenulata*, and *C. famata* had high Niche Overlap Index (NOI) scores, suggesting their ability to successfully assimilate a wide variety of carbon sources, making these nutrients unavailable for fungi and allowing a rapid proliferation of yeasts (that is, competitive exclusion) (Spadaro et al., 2010; Bautista-Rosales et al., 2014). In addition, *S. cerevisiae*, *S. chevalieri*, *C. catenulata*, *C. famata*, *C. sake*, and others produce siderophores that seize ferric iron, which is biologically important as a co-factor in various fungal enzymes (Meziane et al., 2005; Macagnan et al., 2008), making this ion unavailable to filamentous fungi.

Furthermore, natural compounds were tested in the field, and their SR-reducing efficacy was evaluated in a total of six cases. The natural compounds included plant resistance inducers (specifically, COS-OGA, a complex of oligochitosans and oligopectates), zeolite, and a complex Cu-S. All these compounds exhibited high SR-controlling efficacy. For instance, Calzarano et al (2019 and 2020) showed that zeolitite-based products provided >74% SR control, similar to the fungicides cyprodinil and fludioxonil. Nigro et al. (2006) also evaluated the efficacy of some carbonates and bicarbonates when applied 21 and 5 days before harvest. They reported an SR incidence reduction of 59%, which is higher than that obtained by chemical treatments with cyprodinil and fludioxonil. In addition, calcium might show both direct and indirect SR-reducing activities. The direct activity has been demonstrated for *P. expansum* and *B. cinerea*, where in calcium inhibits the polygalacturonase activity that mediates pathogenicity (Droby et al., 1997; Conway et al., 1999). The indirect activity is related to increased resistance of plant cell walls to hydrolytic enzymes produced by the decay microbes via the formation of calcium cross-linkage between pectin polymers (Tobias et al., 1993).

A few of the selected papers assessed the effects of the combined use of BCA and natural substances. Calvo-Garrido et al. (2013) evaluated the effect of BCAs (that is, the yeast *C. sake* and the fungus *Ulocladium oudemansii*), a natural coating product (Fungicover) able to improve *C. sake* survival on grape host tissues, and a resistance inducer (chitosan). They reported that the application of *C. sake* plus Fungicover reduced SR severity with 58.3% efficacy. The application of *U. oudemansii* in the early season and chitosan or *C. sake* plus Fungicover from veraison to harvest led to SR control with 41.7% and 50% efficacies, respectively.

Finally, Gadoury et al. (2023) showed that nighttime application of germicidal ultraviolet at 200 J/m^2^ significantly reduced the SR severity by 80.2% compared to the untreated test, while the fungicide Oxidate 2.0 (27% hydrogen peroxide plus 2% peroxyacetic acid, BioSafe Systems LLC) had an efficacy of 60.2%.

## 4 Discussion

This review focused on 74 papers published between 1986 and 2023, with a higher number of publications in the last two decades. Despite the increased interest in SR, especially in the northern hemisphere, the present review highlighted several knowledge gaps in SR etiology, epidemiology, and control.

### 4.1 Knowledge gaps in SR etiology

Given the complexity of grape microflora (composed of filamentous fungi, yeasts, and bacteria), the selected papers focused on isolating and identifying microorganisms from the affected berries. However, they could not definitively clarify which species are primarily involved in SR etiology. *Pichia*, *Candida*, *Hanseniaspora*, *Gluconobacter*, and *Acetobacter* were the most frequently isolated yeasts and bacteria from the affected berries and were considered the most important SR-inducing agents. However, a wide variability in the SR-related microbial species was observed across the different studies. This variability was attributed to the varying geographic areas and climatic conditions, grape varieties, grape maturity stages, and berry damage status. This variability could also be originated from when the microbial analysis has been conducted in relation to the succession of the different functional groups during the berry rotting processes, the initial presence of yeast producing ethanol from sugars followed by acetic bacteria able to oxidize the ethanol in acetic acid) (Barata et al., 2012b). To better understand the role of microorganisms isolated from the affected grape bunches in the different studies, the isolated microorganisms must be artificially inoculated into healthy to verify the Koch’s postulates. However, such inoculation studies have rarely been conducted. Even more rarely, studies were conducted considering not only the inoculation of single microbial species but their consociation and/or succession, given that the order/succession of microorganisms and their trophic levels have shown a relevant effect in some studies (Dutt et al., 2022).

### 4.2 Inconsistency among artificial inoculation studies

A general inconsistency was observed in the methods used to assess the incidence and severity of SR in vineyards, making inter-study comparisons challenging. Inconsistencies were also found in the methods used for pathogenicity assessment in artificial inoculation studies, which were also related to advancements in research and scientific methods, e.g., from classical microbial analysis to DNA-based microbiome analysis. This observation was significant with respect to extracting robust information on disease etiology. Efforts should then be devoted by researchers to devise standard inoculation methods (taking into account healthy or wounded berries, type of wounding, etc.) with uniform inoculum concentrations. Such studies should be conducted with a common method or scale for assessing disease symptoms, such as discoloration and rotting severity, complemented with the evaluation of chemical changes in the juice of the inoculated berries, such as total and volatile acidity, pH, glycerol, acetic and gluconic acid levels, and alcohol levels, which are essential factors impacting SR progression. Two additional aspects should be considered when planning such studies: The temperature at which inoculated berries are incubated and the time lag between inoculation and assessment of disease and chemical composition. These aspects significantly impact the results. The present review revealed high variability for both these aspects, making inter-study comparisons difficult.

### 4.3 Knowledge gaps in SR epidemiology

The variability in the temperatures employed in the selected artificial inoculation studies revealed another knowledge gap, which is the ecology and epidemiology of the causal agents. We found only one study on the temperature response patterns of SR-inducing microorganisms (Huber, 2016), and none of the studies considered water response as either relative humidity, surface water, or water activity in berry tissue. Furthermore, none of the studies reported the survival rates, inoculum sources, and changes in berry susceptibility depending on growth stages, with the exception of Hall et al. (2018b), who observed that SR symptoms do not appear before the sugar content of berries is 15°Brix. However, some studies thoroughly evaluated the role of insect vectors in the inoculum dispersal, which was different from other possible microbe dispersal mechanisms. Our epidemiological knowledge on SR is less than that on other important grape diseases. This gap markedly impacts the efficacy of SR control methods (Hall et al., 2018b), and further research is warranted to fill this gap.

### 4.4 Inconsistency among SR control studies

The studies selected in this review also provided information on SR management strategies. The management options for SR are limited, and the efficacy trials often result in poor, variable, and inconsistent levels of control, which might be attributed to the lack of knowledge on disease epidemiology. Because of the ongoing increase in restrictions on pesticide use (Pertot et al., 2017), reliable, low-impact alternatives for disease control, such as BCAs, natural substances, and agronomical practices, are urgently required. The different strategies for SR control adopted in the studies selected in this review could be integrated through a network meta-analysis that allows direct comparisons of all the strategies and takes into account all the correlations (Madden et al., 2017).

## Data availability statement

The raw data supporting the conclusions of this article will be made available by the authors, without undue reservation.

## Author contributions

CB: Formal analysis, Methodology, Writing – original draft. VR: Conceptualization, Methodology, Writing – review & editing. GF: Conceptualization, Methodology, Writing – review & editing.
