# Supplementary material for: Knowledge gaps on grape sour rot inferred from a systematic literature review
Source: Front Plant Sci. 2024 Jul 3;15:1415379. doi: 10.3389/fpls.2024.1415379 (PMC11251901; doi:10.3389/fpls.2024.1415379)
Supplement: Supplementary file 1 [file Table_1.docx]

Supplementary Material

Knowledge Gaps on Grape Sour Rot inferred from A Systematic Literature Review

Chiara Brischetto^1^, Vittorio Rossi^1^, Giorgia Fedele^1*^

*** Correspondence:** Giorgia Fedele; [giorgiafedele@unicatt.it](mailto:giorgiafedele@unicatt.it)

# Supplementary table

**Supplementary Table.** Efficacy of different treatments for the management of sour rot in grapes, categorized as low (L: 0–30% reduction of microorganism growth in laboratory), medium (M: 30–60%), and high (H: >60%).

| Biological Control Agents (BCAs) | Pathogen | Field/Laboratory | Efficacy | Reference |
| --- | --- | --- | --- | --- |
| *Kloeckera apiculata* | *Aspergillus niger* | Lab^2^ | M | McLaughlin et al.(1992) |
| *Candida guilliermondii* |  |  | L |  |
| *Saccharomyces cerevisiae* (14)^1^ | *Aspergillus caelatus* | Lab | L | Nally et al. (2013) |
| *S. chevalieri* |  |  | H |  |
| *S. kluyveri* |  |  | L |  |
| *Candida catenulata* (2) |  |  | L |  |
| *C. famata* |  |  | L |  |
| *C. rugosa* |  |  | H |  |
| *C. sake* (4) |  |  | L |  |
| *C. versatilis* |  |  | L |  |
| *Debaryomyces vanrijiae* (2) |  |  | L |  |
| *Dekkera anomala* (2) |  |  | M |  |
| *Issatchenkia orientalis* |  |  | L |  |
| *Kluyveromyces marxianus* (2) |  |  | L |  |
| *Pichia membranifaciens* (2) |  |  | L |  |
| *Sporobolomyces roseus* |  |  | L |  |
| *Torulaspora delbrueckii* (8) |  |  | L |  |
| *S. cerevisiae* (14) | *Aspergillus carbonarius* | Lab | L |  |
| *S. chevalieri* |  |  | L |  |
| *S. kluyveri* |  |  | L |  |
| *C. catenulata* (2) |  |  | L |  |
| *C. rugosa* |  |  | L |  |
| *C. sake* (4) |  |  | L |  |
| *C. versatilis* |  |  | L |  |
| *D. vanrijiae* (2) |  |  | L |  |
| *D. anomala* (2) |  |  | L |  |
| *I.orientali* |  |  | L |  |
| *K. marxianus* (2) |  |  | L |  |
| *P. membranifaciens* (2) |  |  | L |  |
| *S. roseus* |  |  | L |  |
| *T. delbrueckii* (8) |  |  | L |  |
| *S. cerevisiae* (14) | *Aspergillus terreus* | Lab | L |  |
| *S. chevalieri* |  |  | L |  |
| *S. kluyveri* |  |  | L |  |
| *C. catenulata* (2) |  |  | L |  |
| *C. famata* |  |  | H |  |
| *C. rugosa* |  |  | L |  |
| *C. sake* (4) |  |  | L |  |
| *C. versatilis* |  |  | L |  |
| *D. vanrijiae* (2) |  |  | H |  |
| *D. anomala* (2) |  |  | L |  |
| *I. orientalis* |  |  | H |  |
| *K. marxianus* (2) |  |  | H |  |
| *P. membranifaciens* (2) |  |  | L |  |
| *S. roseus* |  |  | L |  |
| *T. delbrueckii* (8) |  |  | L |  |
| BCAs | **Pathogen** | **Field/Laboratory** | **Efficacy** | **Reference** |
| *S. cerevisiae* (14)  *S. chevalieri*  *S. kluyveri*  *C. catenulata* (2) | *Aspergillus versicolor* | Lab | L  L  L  M | Nally et al. (2013) |
| *C. famata*  *C. rugosa*  *C. sake* (4)  *C. versatilis*  *D. vanrijiae* (2)  *D. anomala* (2)  *I. orientalis*  *K. marxianus* (2)  *P. membranifaciens* (2)  *S. roseus*  *T. delbrueckii* (8) | *Aspergillus versicolor* | Lab | L  L  L  L  L  L  L  L  L  L  L |  |
| *Candida intermedia*  *Cyberlindnera jadinii*  *Lachancea thermotolerans*  *Candida friedrichii* | *Aspergillus carbonarius* | Lab | H  L  H  H | Fiori et al. (2014) |
| BCAs and chemicals |  |  |  |  |
| *Aureobasidium pullulans*  Fludioxonil +^3^ Cyprodinil | Sour rot | F^2^ | H  H | Dimakopoulou et al. (2008) |
| Mepanypirim  *Candida saitoana +* Chitosan  *C. saitoana +* antifungal lytic enzyme | Sour rot | F | L  L  L | Schena et al. (2005) |
| BCAs and natural substances |  |  |  |  |
| *C. sake*  *C. sake* plus Fungicover | Sour rot | F | M  L | Carbó et al. (2019) |
| *C. sake* + Fungicover  *C. sake* low + Fungicover  Chitosan  *Ulocladium oudemansii*/^4^Chitosan  *U. oudemansii*/*C. sake* + Fungicover  *C. sake* + Fungicover/Chitosan  Fungicover | Sour rot | F | M  M  L  M  M  M  L | Calvo‐Garrido et al. (2013) |
| Natural substances |  |  |  |  |
| Italian chabasite-rich zeolitite  Italian copper chabasite-rich zeolitite | Sour rot | F | H  H | Calzarano et al. (2020) |
| COS–OGA  COS-OGA + mycorrhiza  Cu–S complex 3%  Cu–S complex 3% + mycorrhiza | Sour rot | F | H  H  H  H | Calderone et al. (2022) |
| Natural substances and chemicals |  |  |  |  |
| Calcium chloride  Sodium bicarbonate  Sodium carbonate  Potassium carbonate  Procymidone | Sour rot | F | H  H  H  M  L | Nigro et al. (2006) |
| Natural substances and chemicals | **Pathogen** | **Field/Laboratory** | **Efficacy** | **Reference** |
| Fludioxonil + Cyprodinil | Sour rot | F | L | Nigro et al. (2006) |
| Fludioxonil + Cyprodinil  Chabasite-rich zeolitites  Fludioxonil + Cyprodinil + zeolitites | Sour rot | F | H  H  H | Calzarano et al. (2019) |
| Chemicals |  |  |  |  |
| Fludioxonil + Cyprodinil  Carbendazim  Cyprodinil | *Aspergillus carbonarius* | F | M  L  L | Tjamos et al. (2004) |
| Fludioxonil + Cyprodinil  Carbendazim  Cyprodinil | *Aspergillus niger* | F | M  L  L |  |
| Fludioxonil + Cyprodinil  Carbendazim  Cyprodinil | Sour rot | F | H  L  L |  |

^1^() = number of strains

^2^ Lab = Laboratory; F= Field

^3^+ = combination of the treatments

^4^/= before slash treatment applied in early season and after slash treatment applied in late season

**References**

Calderone, F., Vitale, A., Panebianco, S., Lombardo, M. F., and Cirvilleri, G. (2022). COS-OGA applications in Organic Vineyard manage major airborne diseases and maintain postharvest quality of wine grapes. Plants (Basel). 11, 1763. doi: 10.3390/plants11131763

Calvo-Garrido, C., Viñas, I., Elmer, P. A. G., Usall, J., and Teixidó, N. (2013). *Candida sake* CPA-1 and other biologically based products as potential control strategies to reduce sour rot of grapes. Lett. Appl. Microbiol. 57, 356–361. doi: 10.1111/lam.12121

Calzarano, F., Seghetti, L., Pagnani, G., and Di Marco, S. (2020). Italian zeolitites in the control of grey mould and sour rot and their effect on leaf reflectance, grape and wine. Agriculture 10, 580. doi: 10.3390/agriculture10120580

Calzarano, F., Valentini, G., Arfelli, G., Seghetti, L., Manetta, A. C., Metruccio, E. G., et al. (2019). Activity of Italian natural chabasite-rich zeolitites against grey mould, sour rot and grapevine moth, and effects on grape and wine composition. Phytopathol. Mediterr. 58, 307–322.

Carbó, A., Torres, R., Usall, J., Marı́n, A., Chiralt, A., and Teixidó, N. (2019). Novel film-forming formulations of the biocontrol agent Candida sake CPA-1: biocontrol efficacy and performance at field conditions in organic wine grapes. Pest Manage. Sci. 75, 959–968. doi: 10.1002/ps.5200

Dimakopoulou, M., Tjamos, S. E., Antoniou, P. P., Pietri, A., Battilani, P., Avramidis, N., et al. (2008). Phyllosphere grapevine yeast *Aureobasidium pullulans* reduces *Aspergillus carbonarius* (sour rot) incidence in wine-producing vineyards in Greece. Biol. Control. 46, 158–165. doi: 10.1016/j.biocontrol.2008.04.015

Fiori, S., Urgeghe, P. P., Hammami, W., Razzu, S., Jaoua, S., and Migheli, Q. (2014). Biocontrol activity of four non- and low-fermenting yeast strains against Aspergillus carbonarius and their ability to remove ochratoxin A from grape juice. Int. J. Food Microbiol. 189, 45–50. doi: 10.1016/j.ijfoodmicro.2014.07.020

McLaughlin, R. J., Wilson, C. L., Droby, S., and Chalutz, E. (1992). Biological control of postharvest diseases of grape, peach, and apple with the yeasts Kloeckera apiculate and Candida guilliermondii. Plant Dis. 76, 470–473. doi: 10.1094/PD-76–0470

Nally, M. C., Pesce, V. M., Maturano, Y. P., Toro, M. E., Combina, M., Castellanos de Figueroa, L. I., et al. (2013). Biocontrol of fungi isolated from sour rot infected table grapes by Saccharomyces and other yeast species. Postharvest. Biol. Technol. 86, 456– 462. doi: 10.1016/j.postharvbio.2013.07.022

Nigro, F., Schena, L., Ligorio, A., Pentimone, I., Ippolito, A., and Salerno, M. G. (2006). Control of table grape storage rots by pre-harvest applications of salts. Postharvest Biol. Technol 42, 142–149. doi: 10.1016/j.postharvbio.2006.06.005

Schena, L., Nigro, F., Soleti Ligorio, V., Yaseen, T., Ippolito, A., and El Ghaouth, A. (2005). Biocontrol activity of bio-coat and biocure against postharvest rots of table grapes and sweet cherries. Acta Hortic. 682, 2115-2120. doi: 10.17660/ActaHortic.2005.682.288

Tjamos, S. E., Antoniou, P. P., Kazantzidou, A., Antonopoulos, D. F., Papageorgiou, I., and Tjamos, E. C. (2004). *Aspergillus Niger* and *Aspergillus carbonarius* in Corinth raisin and wine-producing vineyards in Greece: population composition, ochratoxin A production and chemical control. J. Phytopathol. 152, 250–255. doi: 10.1111/j.1439– 0434.2004.00838.x
